# Open abdomen in trauma patients: a double-edged sword

**DOI:** 10.1186/s40779-016-0079-0

**Published:** 2016-04-01

**Authors:** Yu-hua Huang, You-sheng Li

**Affiliations:** Department of General Surgery, Jinling Hospital, Nanjing University School of Medicine, 305 East Zhongshan Road, Nanjing, 210002 China

**Keywords:** Open abdomen, Abdominal compartment syndrome, Damage control, Enteroatmospheric fistula, Negative pressure therapy

## Abstract

The use of open abdomen (OA) as a technique in the treatment of exsanguinating trauma patients was first described in the mid-19^th^ century. Since the 1980s, OA has become a relatively new and increasingly common strategy to manage massive trauma and abdominal catastrophes. OA has been proven to help reduce the mortality of trauma. Nevertheless, the OA method may be associated with terrible and devastating complications such as enteroatmospheric fistula (EAF). As a result, OA should not be overused, and attention should be given to critical care as well as special management. The temporary abdominal closure (TAC) technique after abbreviated laparotomy was used to improve wound healing and facilitate final fascial closure of OA. Negative pressure therapy (NPT) is the most commonly used TAC method.

## Introduction

The use of open abdomen (OA) as a technique to manage exsanguination of trauma patients was first described by Ogilvie [1] in 1940 during World War II, but not much attention was paid to it until the 1980s. In 1983, Stone et al. [2] reported that abbreviated laparotomy with abdominal tamponade using sponges and with the abdomen left open was an effective technique to control organ injury and improve the survival rate. Later, Rotondo noted that repairing all the injuries no longer benefited patient with multi-organ and severe vascular injuries, and the term “damage control” was put forth in 1993 [3]. Since that time, the OA approach has been used for both military and civilian trauma cases and has undergone significant development. In addition, OA use is not limited to trauma and has been extended to the management of emergency general surgery, vascular surgery, intra-abdominal sepsis and acute pancreatitis [4]. During the initial operation and resuscitation, the presence of the lethal triad of hypothermia, acidosis and coagulopathy may contribute to high mortality in major abdominal trauma patients. The concept of OA – not closing the abdominal fascia during the initial laparotomy – operates under the principle of damage control, which emphasizes the importance of an abbreviated laparotomy focused on rapid control of hemorrhage and gastrointestinal contamination without extensive procedures on physiologically unstable patients [5]. After laparotomy, the abdomen is temporarily closed by a temporary abdominal closure (TAC) technique without formal fascial approximation in the midline. The definitive surgery along with the attempt to close the abdomen is performed later, only after the patient’s physiology is normalized [6].

Although OA is life-saving for some trauma patients, these patients are faced with a number of problems and challenges. The high risk of enteroatmospheric fistula (EAF) formation, for which the mortality rate is still as high as 40 % [7], is the biggest problem. Moreover, fluid loss due to an open peritoneal cavity, electrolyte abnormalities, and the large ventral hernia should also be taken into consideration. The management of the large open wound, the goal to definitively close the abdomen, and the specialized nutrition support are all challenges challenge for doctors and nurses.

In this review, we present a perspective on the indications, advantages and complications of OA in critically ill trauma patients. The prevention and management of complications are also included.

## Who needs the OA technique?

Up to 25 % of trauma laparotomies need OA management [8]; however, OA should not be abused because of its significant complications. There is a lack of a systematic approach in the management of such a serious surgical problem. The decision to use OA and TAC is not standardized; it is still dependent on the individual surgeon and his or her experience, so the OA may not prove beneficial for patients in some case series [9]. The following are common indications for OA in trauma patients.

### Abdominal Compartment Syndrome (ACS)

ACS is a lethal complication of intra-abdominal hypertension (IAH) in trauma patients and is defined by the World Society of the Abdominal Compartment Syndrome (WSACS) as an intra-abdominal pressure (IAP) of >20 mmHg with clinical signs of new organ dysfunction/failure [10]. IAH is defined by a sustained or repeated pathological elevation of IAP >12 mmHg. In 2013, the WSACS recommended an IAH grading system from I to IV to indicate increasing severity of IAH [11]. The prevalence of ACS in major trauma patients is approximately 30 %, with the mortality rate as high as 60 % [12]. Post-injury ACS has been an independent risk factor for death in critically ill trauma patients and is classified into primary ACS (associated with injury or disease in the abdominopelvic region) and secondary ACS (not originating from the abdominopelvic region) [11]. The essential reasons for multi-organ dysfunction in ACS are the decreased organ perfusion and compromised venous return due to increasing intra-abdominal pressure [13].

Surgical decompression by OA is a standard treatment for ACS. After decompression, management of the OA is continued with temporary closure, and frequent monitoring of IAP is essential, as subsequent massive resuscitation can cause recurrent ACS even with OA [14]. Aside from already identified cases of ACS, when the IAP ≥25 mmHg and ACS is likely, decompressive laparotomy and OA should also be considered [4].

### Damage control

Over the past few years, damage control has evolved and is used widely as an acceptable technique to manage abdominal emergencies in trauma patients [15]. OA is critical to damage control because the continuous fluid resuscitation in conjunction with abdominal packing can lead to ACS. For example, in cases of abdominal penetrating or blunt injury involving hepatic, non-hepatic, or vascular injuries with intra-abdominal packing, the use of OA should be considered, and an early decision to truncate a definitive operation should be made as soon as possible [4]. Recently, Steven et al. [16] found that trauma patients in need of a damage control laparotomy who develop acidosis, coagulopathy, and hypothermia are at risk of IAH and ACS and require OA. The final stage of damage control lies in abdomen closure, but the patients must be selected carefully to prevent the occurrence of IAH [17].

### Intra-abdominal infection

Infection and sepsis-associated complications are major causes of late mortality for trauma patients. When a single laparotomy is not enough to control the source of infection, OA remains a mainstay therapy to ease return to the peritoneal cavity, removal of necrotic tissue and effective drainage [18]. However, given that the potential complications of OA may negate its benefit in controlling infection, this option should not be overused [19].

## Benefits of OA

Several studies have shown improved mortality in severely injured patients treated with damage control laparotomy; this improved mortality is thought to be associated with better ICU care and increased OA use [20]. The OA is considered an important method to treat physiologically unstable trauma patients, and its benefits have been well recognized. The OA technique helps to prevent IAH and ACS [21] and avoids all the problems of closure under tension. Decompression can frequently lead to good physiological response, and the reported survival rates range from 33 % to as high as 100 % [22]. These positive results are due to decreased intra-abdominal pressure and improved organ oxygen supply. Despite the initial good result, the outcome may be still poor in some cases because of the reperfusion injury with delayed decompression. With earlier recognition and immediate OA, the patients who are decompressed within 24 h reach higher survival rates than those who are decompressed after 48 h [12].

After restoration of the physiologic envelope in trauma patients, planned or on-demand re-laparotomy may be necessary for additional surgical procedures or for infection control in stable patients. The OA technique enables repeated access to the peritoneal cavity for re-operation and repeated debridement of nonviable tissue and infected peritoneal fluid both in the operating room and at the bedside [23]. Furthermore, OA facilitates the damage control procedure by avoiding the step of re-opening the abdomen, and the patient is free from a second surgical procedure.

In addition, OA preserves the unviolated abdominal fascia for subsequent closure [14] because the muscle and fascial layers do not need to be sutured and incised again, thereby minimizing the damage to the fascia. The earlier identification of intra-abdominal complications and accurate judgment of infection and inactive intestinal tract secondary to trauma can also be achieved in OA patients.

## Major complications of OA

### EAF

EAF is defined as the occurrence of a fistula in the exposed bowels of an OA patient, which leads to an abnormal communication between the intra-abdominal gastrointestinal tract and atmosphere [24]. The incidence of EAF in trauma patients with OA ranges from 2 to 42.4 % [25]. A higher rate of EAF occurrence (12.1 %) is observed in septic OA than in non-septic OA (3.7 %) patients [26]. It is well recognized that the possibility of EAF development increases as the time from damage control surgery to definitive abdominal closure is prolonged [27].

The etiologies of EAF are various. OA forces the bowels to be exposed to an open environment and therefore puts them under the danger of fistula formation. The conditions necessitating the surgery can increase the risk of fistula; these include persistent abdominal infection, anastomotic leakage, ongoing bowel ischemia, and distal bowel obstruction [27]. EAF arises as a result of adhesion of the bowels to themselves or to the fascia because of inappropriate management. The use of a synthetic mesh for TAC and bridge repair is also related to EAF formation. Polypropylene meshes have been shown to lead to unacceptably high rates of fistula formation [28]. Furthermore, frequent complicated abdominal dressing changes and re-operation may lead to mechanical injury to the bowels and cause fistula [24]. A multicenter prospective study found that large bowel resection, large volume fluid resuscitation, and an increasing number of abdominal re-explorations were significant predictors of EAF in patients with OA [29].

EAF is regarded as one of the most devastating complications of OA and is a nightmare for patients, with a reported mortality of over 40 % [30]. The absence of a fistula tract and the lack of well-vascularized surrounding tissue are specific characteristics of EAF [31]. As a result, the spontaneous healing of EAF is nearly impossible. Location and fistula output are factors crucially affecting the prognosis and spontaneous closure of the EAF. Distal and low-output EAFs may have a high spontaneous closure rate [32]. The unhealed EAF has to be treated by re-operation, which delays the closure of OA and increases the risk of developing other complications. Moreover, as re-operations are more frequent after OA, new EAF formation can occur, thus creating a vicious cycle.

The location of EAF within an OA results in spillage of enteric content directly into the open peritoneal cavity and causes uncontrolled infection and intra-abdominal abscess, especially in deep and high-output fistula. The absorption of a large amount of toxic and infected fluid by the peritoneum leads to sever sepsis and even septic shock as well as multi-organ dysfunction [33], which significantly increases the mortality rate. Therefore, deep EAF is considered a surgical emergency because of the ongoing peritonitis and should be managed immediately [32]. Additionally, persistent infection usually contributes to the formation of dense adhesions between the peritoneum and the viscera, increasing the difficulty of subsequent operations and the risk of damage to the bowels. Edema of the bowels is also observed as a result of abdominal infection, leading to paralytic ileus and ACS. Superficial EAF drains on top or to the side of the granulating abdominal wound [27] and causes septic wound complication, resulting in the fascial closure becoming difficult and even impossible.

The continued loss of intestinal fluids rich in electrolytes through an EAF causes electrolyte and acid–base disturbances and worsens dehydration because of excessive fluid loss from the exposed surface of OA. Furthermore, all or some of the proteins reabsorbed by the small bowel are likely to be lost through the fistula [33]. This loss, along with protein loss from the OA itself, hypercatabolism associated with underlying disease, and decreased uptake of food, results in patients with EAF usually presenting with hypoalbuminemia and malnutrition. This ultimately puts patients under the risk of severe infection and negatively influences their recovery. Malnutrition has become a leading cause of death in patients with fistula.

### Chronic/planned ventral hernia

OA patients carry a potential risk of ventral hernia formation. The incidence of chronic ventral hernia ranges from 13 to 80 %, depending on the patient’s individual characteristics and the different institutional practices [34]. Once it has been determined that the abdominal fascia will not come together during the first hospitalization because of fistula formation, large fascial defect and loss of abdominal wall tissue, a planned ventral hernia is considered [35]. The use of a split-thickness skin graft and skin flap can result in ventral hernia [36]. Though these patients may have the hernia repaired by abdominal wall reconstruction during the second hospitalization, the planned ventral hernia has a negative impact on them both physically and psychologically. A recent guideline has developed an organized evidence-based approach to the management of the elective repair of the planned ventral hernia [37]. The recurrence rates of hernia vary depending on the method of repair and the length of follow-up. A 15-year follow-up study demonstrated a recurrence rate of 14 % for all methods, and the modified components separation technique without prosthetics was associated with a lower rate of hernia recurrence (5 %) [38]. Large ventral hernias may be associated with prolonged recovery due to physical discomfort or loss of function [34]. If the ventral hernia is complicated by the presence of fistula, even worse outcomes may occur, which limit the patients’ functional status [39]. Studies have observed that patients with large chronic ventral hernias show persistent significant impairment of activity, productivity, and quality of life [40].

## Management of OA and its complications

### Temporary closure by negative pressure therapy (NPT)

Regardless of the type of complication, the prolonged exposure of the peritoneal viscera with no or inappropriate coverage is the root. The final goal of management of OA is achieving definitive fascial closure without potential risk of ACS to prevent complications. In these circumstances, the TAC technique is necessary. However, clinical experience demonstrates that just coverage of the exposed viscera by traditional TAC is no longer sufficient. At present, NPT has become a standardized method, and also the most extensively used method for TAC, because of its advantages in facilitating final abdominal closure. The first generation of NPT was reported by Brock et al. [41] and was called Vacuum Packing (VP). In the VP technique, a fenestrated polyethylene sheet is placed beneath the peritoneum and covers all the peritoneal viscera. Then, a moist sterile surgical towel is placed over the polyethylene sheet and folded to fit inside the wound. Next, two 10-French flat silicone drains are put on the top of towel and connected to the suction source. Finally, the whole wound is sealed with an adhesive polyester drape. This design is also called a “sandwich;” the inner layer of polyethylene sheet serves as a barrier and prevents the adhesion, the drains apply the negative pressure, and the towel and suction allow the remove of toxic peritoneal fluid [42]. In addition, it also allows for safe movement and prone positioning of the patients [4]. The VP has led to an evolution in the management of OA, with a high rate of primary fascial closure [43, 44] and a low rate (5 %) of complications with intestinal fistula formation [45]. VP has been commercialized by KCI as the Vacuum Assisted Closure (VAC) Abdominal Dressing System. The VAC system replaces the absorptive towel in the middle layer of VP with an absorptive polyurethane sponge [23]. The degree of negative pressure is controlled by an external computerized vacuum device, and fluid is collected in a container [22]. The VAC system does well in promoting wound healing, reducing bowel edema and approximating the edges of the fascia, therefore facilitating the final closure of abdominal wall. For trauma patients with OA, VAC is the most commonly used technique and has the highest delayed primary closure rates [46]. A prospective study reported the definitive fascial closure rate to be as high as 88 %, with a mean time to closure of 9.5 days [47]. When managing OA with VAC and mesh-mediated fascial traction, the final abdominal closure rate rose to 89 and 93 % in three separate studies [48–50]. A new generation of NPT, the ABThera system, has also been developed [51], and it has the added advantage that the inner sponge has six extensions that can extend to the ends of the plastic sheet to facilitate more effective evacuation of peritoneal fluid [52]. Compared with VP, the ABThera system has higher 30-day primary fascial closure rates and lower 30-day all-cause mortality [53]. Nevertheless, some points need to be kept firmly in mind when using NPT. Firstly, the negative pressure should be set individually, in that high negative pressure may aggravate bleeding. Secondly, polyurethane foam should never be placed directly in contact with the bowels; a non-adherent layer is always between them. Thirdly, bladder pressure is monitored regularly for development of ACS [51].

### Management of EAF

The management of EAF remains a great challenge, and the most important principle is prevention. The exposed bowels should be protected by a covering of a biologic mesh or other non-adherent material, and exposed viscera must not come into direct contact with the non-absorbable mesh. Dressing changes and re-entry into the peritoneal cavity should be limited to experienced care providers who are familiar with the wound [54]. Finally, surgeons should always keep in mind the goal of achieving definitive fascial closure to shorten the exposure time [27].

However, prevention is not always achieved. Faced with the occurrence of EAF, source control and elimination of ongoing contamination of the peritoneal cavity and wound are essential for survival. The best solution to control the effluent is exteriorization of the fistula and surgical intestinal diversion proximal to a distal fistula [54]. If proximal diversion is not possible because of bowel edema and mesenteric shortening, converting the EAF to an enterocutaneous fistula is considered. It can divert the fistula effluent and develop a fistulous tract [55]. The isolation of intestinal content by a “floating stoma” can also be performed. It is accomplished by suturing the edges of the holes in the gut to the plastic silo used for temporary coverage. In this way, a controlled fistula resembling a stoma is formed, and source control is achieved. Importantly, the ongoing insult to the peritoneal cavity is stopped until the granulation of the viscera allows for skin grafting [56]. Appropriate use of NPT can also achieve effective control of the spillage of intestinal contents and protection of surrounding tissues and exposed bowels [57]. Tavusbay and colleagues [58] reported the treatment of seventeen patients with EAF by NPT with an average duration of 43.6 days. Of the patients, four patients achieved nonsurgical spontaneous closure of the fistulae, and six patients had final successful definitive fascial closure, while eight patients (44.4 %) died due to intra-abdominal infections and sepsis. No NPT-associated complications were observed. Another case report also showed the advantages of isolation techniques by NPT in managing wounds with EAF [59]. A modification of NPT called the “baby bottle nipple method” has been designed; it is said to have the potential to provide both control of the fistula effluent and accurate measurement and collection of the subsequent output [22, 60]. On rare occasions, biological repair materials, such as fibrin glue, human acellular dermal matrix (HAMD) and split-thickness skin graft, are used to seal the EAF; however, they are only suitable for small and low-output fistulas, and their success rate is low [24].

The correction of fluid and electrolyte imbalances is critical for these patients. The amount of supplied fluid should cover the estimated fistula output as well as losses due to fever and sepsis [6, 27]. Likewise, replacement fluid selection should be based on the electrolyte composition and tonicity of the intestinal fluid losses [6]. Receiving enough nutrition, but not being overfed, is another important point for patients. Caloric and protein requirements in patients with a high-output fistula may reach 30 kcal/kg/d and 1.5–2.5 g/kg/d, respectively [61]. The conventional route for nutrition support is parenteral nutrition according to the concept of “bowel rest.” However, enteral nutrition should be administered in appropriate stable patients despite its potential difficulty and challenges [22]. Jianyi et al. [33] have studied the effect and safety of EN in nine patients with EAF. The results were satisfactory in that EN was successfully implemented in all patients, and all of them were liberated from PN upon hospital discharge; only one patient had a feeding-associated complication (ileus). The results showed that EN could be safely implemented in EAF patients; however, research on a larger population is required. In addition, the length and function of residual bowel segments should be assessed to ensure adequate absorption of enteral nutrients and avoid ileus due to bowel immobility [33].

The ultimate surgical correction with fistula resection should be delayed until the patients are clinically stable, of good general status, and free of infection. The final surgery often requires complex abdominal wall reconstruction, which may be 6–12 months after the initial laparotomy [32].

## Conclusions

The list of indications for use of OA in trauma patients has expanded from life-saving decompression of ACS and damage control to intra-abdominal sepsis secondary to trauma. OA has become an important tool for managing physiologically unstable patients requiring urgent abdominal surgery. However, OA-associated complications are a still great burden and present challenges both for the surgeons and patients; of these complications, EAF is most devastating. The prevention of EAF is essential. Effluent control and nutrition support are key points in managing a developed EAF. As definitive abdominal closure without occurrence of ACS is fundamental to reduce complications, NPT has become a popular TAC technique due to its high fascial closure rate.

## Abbreviations

ACS: abdominal compartment syndrome
EAF: enteroatmospheric fistula
HAMD: human acellular dermal matrix
IAH: intra-abdominal hypertension
IAP: intra-abdominal pressure
NPT: negative pressure therapy
OA: open abdomen
TAC: temporary abdominal closure
VAC: vacuum assisted closure
VP: vacuum packing
WSACS: world society of the abdominal compartment syndrome
